# Atypical High-Burden Breakthrough Varicella: A Diagnostic Pitfall in the Two-Dose Vaccine Era

**DOI:** 10.7759/cureus.104369

**Published:** 2026-02-27

**Authors:** Smaran Marupudi, Jared Hensley, Robert Vezzetti

**Affiliations:** 1 Medical Education, University of Texas Rio Grande Valley School of Medicine, Edinburg, USA; 2 Emergency Medicine, Driscoll Children’s Hospital Rio Grande Valley, Edinburg, USA

**Keywords:** cellulitis, chickenpox, exanthema, primary health care, scabies

## Abstract

We present the case of a seven-year-old fully immunized male who developed an intensely pruritic, generalized maculopapular rash that progressed to vesicles. The patient’s rash was initially diagnosed by his primary care provider as possible scabies and treated with topical permethrin. Our examination revealed a diffuse maculopapular and vesicular exanthem with lesions at various stages of evolution, eventually progressing to secondary cellulitis. The patient’s presentation, consistent with varicella (chickenpox), highlights the diagnostic challenges of breakthrough varicella infection and potential complications in fully vaccinated children. Thus, we highlight the importance of having a high clinical index of suspicion for varicella, even in fully immunized children.

## Introduction

Varicella, colloquially known as chickenpox, is an airborne disease caused by primary infection with the varicella-zoster virus, a double-stranded DNA virus of the Herpesviridae family. The illness typically progresses through three sequential stages: (1) an incubation period of 10-21 days, (2) a brief prodrome characterized by low-grade fever and malaise, and (3) onset of the exanthem. The rash evolves rapidly, progressing through macular, papular, vesicular, pustular, and crusting stages within approximately 24-48 hours. New lesions appear in successive waves, creating the characteristic finding of lesions in varying stages of development. Classic lesions appear as clear vesicles on an erythematous base, often described as “dew drops on a rose petal” [1]. Treatment in children is primarily supportive, with antiviral therapy reserved for those at increased risk, such as children over age 12 years and those with chronic skin or lung disease [2]. Antiviral therapy has been shown to reduce symptom severity when initiated within 24 hours of rash onset [1,2].

Before vaccine introduction, varicella was a near-universal childhood disease in the United States, resulting in approximately four million annual cases with an estimated 11,000-13,500 hospitalizations and 100-150 deaths per year [3]. The live-attenuated vaccine was licensed in 1996, leading to a 90% reduction in disease incidence and substantial declines in hospitalizations and mortality by 2006 [3]. Despite these successes, breakthrough infections continued to occur among vaccinated populations, prompting the creation of the two-dose vaccine schedule in 2006 [4,5]. Following implementation of the two-dose regimen, varicella incidence declined by more than 80%, with the greatest reductions observed among school-aged children [3], accompanied by smaller and shorter outbreaks [6].

Despite two-dose varicella vaccination coverage among US children approaching 86-100% by age seven [7], unvaccinated and partially vaccinated individuals continue to account for the majority of varicella cases at the population level [6]. In highly vaccinated settings, however, a greater proportion of reported cases may occur as breakthrough infections [8,9]. Unlike classic varicella, these cases typically present with fewer lesions, minimal systemic symptoms, and shorter disease duration [9]. We present an unusual case with high clinical suspicion for varicella infection in a fully vaccinated child, highlighting the evolving diagnostic challenges of varicella in the post-vaccine era.

## Case presentation

**Figure 1:**
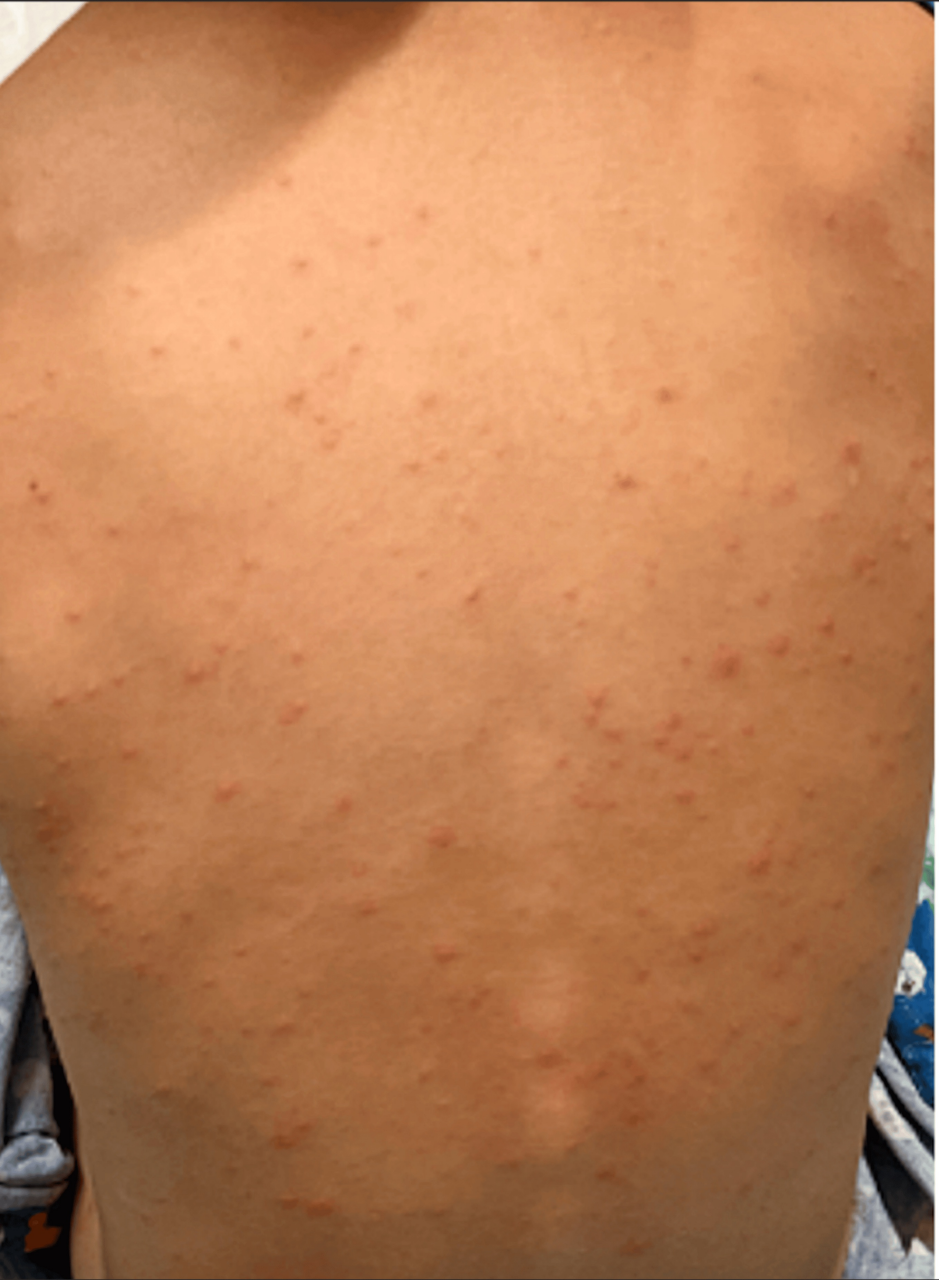
Posterior trunk involvement Diffuse erythematous maculopapular rash with vesicular and crusted lesions at various stages of healing

**Figure 2:**
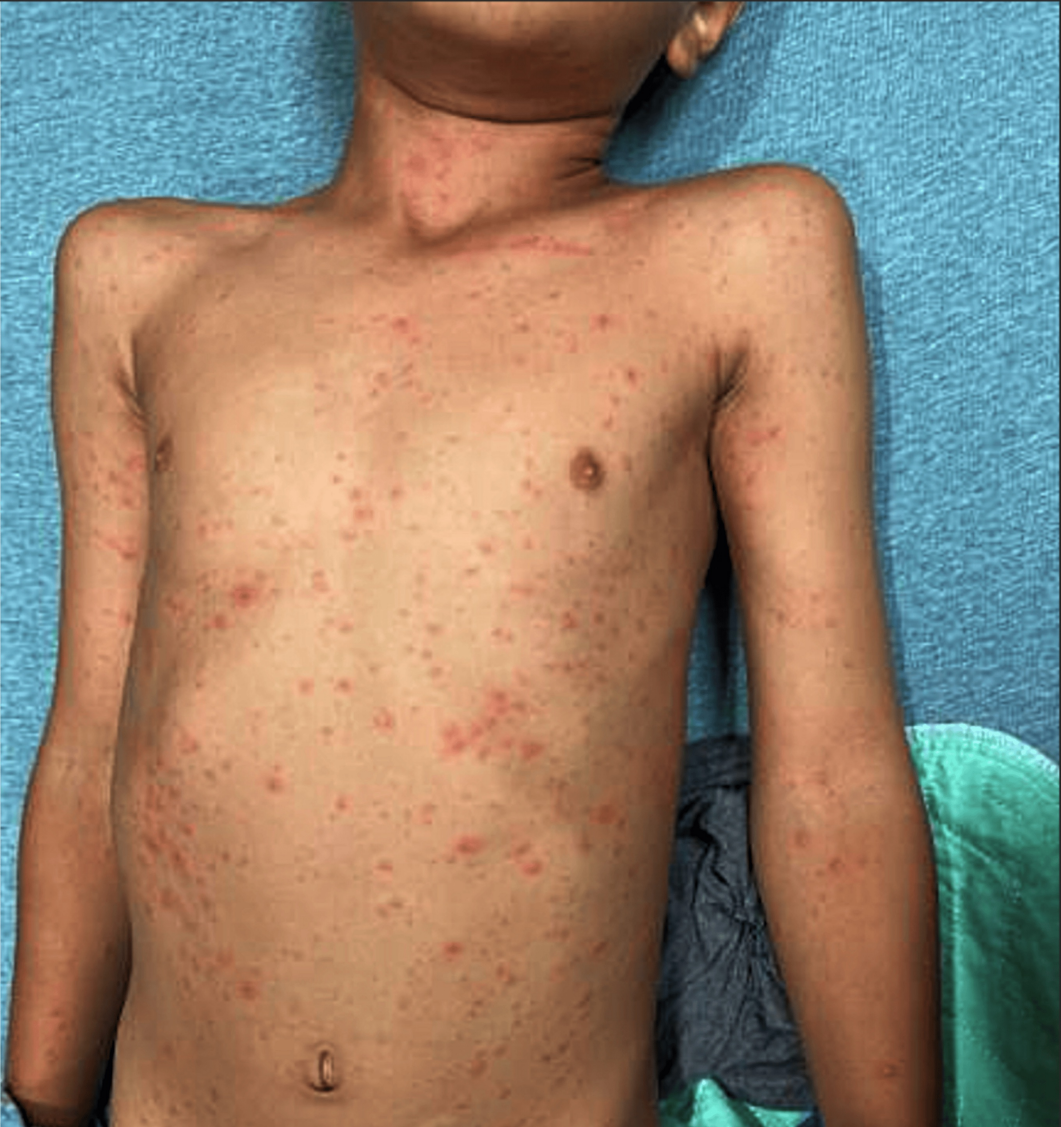
Anterior trunk involvement Widespread erythematous maculopapular rash involving the anterior trunk, with numerous discrete papules and a generalized distribution

**Figure 3:**
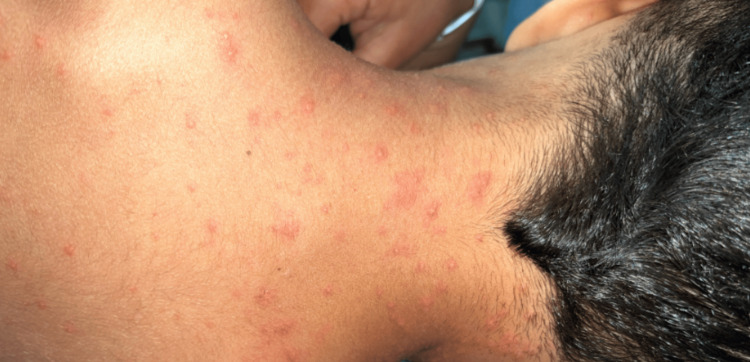
Posterior neck involvement Vesicular lesions on an erythematous base involving the posterior neck

A seven-year-old previously healthy male presented to the pediatric emergency department with a chief complaint of a rash. Three days earlier, the parents had noticed pruritic erythematous papules beginning on the patient’s trunk. The patient’s primary care physician diagnosed the rash as scabies and prescribed topical permethrin. Over the next several days, the rash spread diffusely, and vesicles developed, some of which were noted by the child’s mother to have ruptured, releasing clear fluid, and subsequently crusted. The child had no fever, respiratory, gastrointestinal, or systemic symptoms leading up to the illness. There was no history of new exposures, sick contacts, or recent travel. He was fully immunized, receiving the varicella vaccine at one and four years of age. Upon examination, the patient was in no distress, afebrile, and well-appearing. Skin examination revealed a diffuse erythematous maculopapular rash with hundreds of vesicular and crusted lesions in various stages of healing (Figures 1-3). Initial differentials included varicella, scabies, and vesicular viral exanthems, including those caused by coxsackieviruses. Further examination revealed no linear burrows, petechiae, purpura, target lesions, mucosal lesions, or ocular lesions, further raising suspicion for breakthrough varicella. Given the patient's moderate lesion burden and continued vesicle evolution, a five-day course of oral acyclovir (200 mg/5 mL suspension) was administered following discussion with the family. He was also prescribed daily cetirizine and as-needed hydroxyzine for breakthrough nocturnal pruritus. He was subsequently discharged. The following evening, the patient returned to the emergency department because his parents noticed that his leg lesions were turning dusky. Physical examination revealed areas of diffuse erythema, warmth, and tenderness over the bilateral lower extremities. As secondary bacterial infections are a common complication of varicella, cellulitis was suspected. The patient was prescribed cephalexin 250 mg with instructions to return if his condition worsened. The patient completed his courses of antimicrobial therapy and did well.

## Discussion

The finding of maculopapular rash with vesicles at multiple stages of evolution was suggestive of varicella infection. The American Academy of Pediatrics and Centers for Disease Control and Prevention recommend a two-dose varicella vaccine regimen at ages 12-15 months and four to six years, which provides robust protection, exhibiting efficacies between 93% and 98% for any disease severity and >97% for moderate/severe disease [10-13]. However, “breakthrough” cases still occur in children who are two-dose recipients, with one meta-analysis showing rates of 2.2 cases per 1,000 person-years [14]. Breakthrough cases are defined as varicella in a person vaccinated more than 42 days before the onset of a rash [9,15]. These patients have a modified presentation, exhibiting <50 lesions in a predominantly maculopapular form, less severe systemic symptoms, and overall shorter duration of illness than those who are unvaccinated [8,15,16].

Previously reported definitions for classic and breakthrough disease exist to characterize severity (<50 lesions: mild, 50-500 lesions: moderate, and >500 lesions: severe) [17,18]. Breakthrough cases presenting with 200-300 lesions (moderately severe varicella) are unusual. In the United States, the adoption of the two-dose varicella vaccine regimen has resulted in a significant drop in the incidence and severity of primary varicella, shifting caseloads to a greater proportion of varicella cases being breakthrough presentations [8,10,18]. This epidemiologic change among two-dose recipients has decreased clinician familiarity with an unmodified varicella presentation [16]. Decreased exposure can increase the risk of misdiagnosis, dropping clinical diagnosis sensitivity by 15% when identifying vaccine-modified (breakthrough) cases [19].

Varicella is generally mild in healthy children, with most recovering without complications. However, while rare among healthy children, serious complications have been reported in this age group, with the most common being bacterial skin and soft tissue infections, followed by neurological, respiratory, and gastrointestinal complications [20]. As a result, the increasingly atypical and often mild presentation of varicella may go unrecognized or untreated, delaying diagnosis and, in severe cases, appropriate intervention. Therefore, timely recognition and accurate diagnosis of varicella are essential to prevent progression to severe complications.

The presence of >250 lesions and lack of fever in this seven-year-old child creates a case that has mixed features of breakthrough and primary varicella. The combination of a high-lesion load and intense pruritus in a fully vaccinated child may fall outside the expected post-vaccine breakthrough pattern, increasing the likelihood of misdiagnosis. This case demonstrates how high-lesion breakthrough varicella can mimic a primary varicella presentation that many clinicians no longer routinely encounter in the two-dose vaccine era, creating a meaningful diagnostic pitfall.

Limitations

Limitations of this case include a lack of laboratory confirmation of varicella, the single-case design, and limited follow-up after cellulitis.

## Conclusions

The two-dose vaccine era has led to a marked decline in the incidence of primary varicella and a corresponding shift toward a greater proportion of cases being breakthrough in nature, resulting in clinician unfamiliarity with the full clinical presentation of varicella. This case emphasizes the importance of recognizing an atypical high-lesion (>250 lesions) presentation of breakthrough varicella mimicking primary varicella in pediatric patients. As demonstrated here, a high-lesion presentation can easily be misdiagnosed as scabies or other exanthems. Having a high index of suspicion for atypical breakthrough presentations, even in fully immunized children, is essential for timely prevention of diagnostic delay and subsequent complications.
